# Audiovisual correspondence facilitates the visual search for biological motion

**DOI:** 10.3758/s13423-023-02308-z

**Published:** 2023-05-25

**Authors:** Li Shen, Xiqian Lu, Ying Wang, Yi Jiang

**Affiliations:** 1 State Key Laboratory of Brain and Cognitive Science, CAS Center for Excellence in Brain Science and Intelligence Technology, Institute of Psychology, Chinese Academy of Sciences, 16 Lincui Road, Chaoyang District, Beijing, 100101 China; 2 Department of Psychology, University of Chinese Academy of Sciences, Beijing, 100049 China; 3 Chinese Institute for Brain Research, Beijing, 102206 China

**Keywords:** Biological motion, Crossmodal, Audiovisual integration, Temporal correspondence, Visual search, Attention

## Abstract

Hearing synchronous sounds may facilitate the visual search for the concurrently changed visual targets. Evidence for this audiovisual attentional facilitation effect mainly comes from studies using artificial stimuli with relatively simple temporal dynamics, indicating a stimulus-driven mechanism whereby synchronous audiovisual cues create a salient object to capture attention. Here, we investigated the crossmodal attentional facilitation effect on biological motion (BM), a natural, biologically significant stimulus with complex and unique dynamic profiles. We found that listening to temporally congruent sounds, compared with incongruent sounds, enhanced the visual search for BM targets. More intriguingly, such a facilitation effect requires the presence of distinctive local motion cues (especially the accelerations in feet movement) independent of the global BM configuration, suggesting a crossmodal mechanism triggered by specific biological features to enhance the salience of BM signals. These findings provide novel insights into how audiovisual integration boosts attention to biologically relevant motion stimuli and extend the function of a proposed life detection system driven by local kinematics of BM to multisensory life motion perception.

## Introduction

Vision and audition are two primary sensory modalities that work in concert to guide our attention in the dynamic world (Driver & Spence, 1998; Santangelo & Spence, 2007; ten Oever et al., 2016). For example, in a visual search task, a transient auditory cue synchronized with an abrupt change in a predefined visual target could facilitate the search performance, mostly reflected by faster reaction time (Chamberland et al., 2016; Gao et al., 2021; Van der Burg et al., 2008; Zou et al., 2012). Studies using a spatial cueing task further demonstrated that the tone-induced attentional facilitation in the visual search was independent of top-down task demands, pointing to a stimulus-driven mechanism whereby synchronous audiovisual cues enhance the salience of sensory events to capture attention (Matusz & Eimer, 2011; Turoman et al., 2021). Most of these findings are based on artificial, nonbiological stimuli and demonstrated a crossmodal effect elicited by synchronized but transient audiovisual events. However, relatively little is known about the audiovisual attentional facilitation for natural, biologically relevant stimuli, which usually have continuous and complex dynamic profiles and are likely to involve distinct mechanisms from those for nonbiological ones.

Biological motion (BM) is one of the naturally occurring dynamic stimuli of great evolutionary significance. It has a unique kinematic profile caused by muscle activity under the constraint of gravity (Vallortigara & Regolin, 2006; Wang et al., 2022). Empirical evidence has suggested that the visual processing of BM signals is modulated by the corresponding sounds based on spatial, temporal, or semantic congruency (Brooks et al., 2007; Mendonça et al., 2011; Meyer et al., 2013; Saygin et al., 2008; Schouten et al., 2011; Thomas & Shiffrar, 2010, 2013; van der Zwan et al., 2009; Wuerger et al., 2012). More importantly, the perception of audiovisual temporal relations for BM differs from that for inverted BM that lacks the characteristic kinematic features but maintains low-level properties of normal BM (Saygin et al., 2008), and the temporal window of perceptual audiovisual synchrony is broader for BM than for artificial motion with constant speed (Arrighi et al., 2006). These findings raise the possibility that, different from the processing of non-BM stimuli, a specialized mechanism drives the processing of temporally congruent audiovisual BM cues and may yield a crossmodal attentional facilitation effect.

Notably, the specificity of BM perception arises mainly from the processing of two critical BM cues—namely, the global configuration cue representing the skeletal structure of static bodies and the local motion cue capturing the moving traces of critical joints (Hirai & Senju, 2020; Troje & Westhoff, 2006; Wang et al., 2010). Early studies suggest that global configuration cues are essential to BM perception, as observers can spontaneously recognize moving human figures from visual displays without local image motion (Beintema & Lappe, 2002; Bertenthal & Pinto, 1994). Nonetheless, recent findings highlight the pivotal role of local motion cues in visual BM perception. Above all, humans and some other species (e.g., chicks) exhibit an innate preference toward visual BM over non-BM signals, even when the BM stimuli had no identifiable global configuration but only local kinematic cues (Bardi et al., 2011; Simion et al., 2008; Vallortigara et al., 2005). Moreover, individual variations in the ability to discriminate locomotion direction from the local kinematic cues are genetically determined (Wang et al., 2018), and such abilities stem from one’s sensitivity to the characteristic acceleration patterns carried by the feet motion (Chang & Troje, 2009; Troje & Westhoff, 2006). These findings hint at the existence of a neural mechanism selectively tuned to local BM information in the primitive brain system, which may serve as a ‘life detector’ for legged vertebrates (see reviews by Hirai & Senju, 2020; Lemaire & Vallortigara, 2022; Troje & Chang, 2023). While this ‘life detector’ responds to the dynamics of visual BM stimuli, whether it is sensitive to the temporal correspondence of auditory and visual BM signals and therefore causing a crossmodal attentional facilitation effect remains unexplored.

In the current study, we investigated whether and how concurrent auditory signals influence the selective attention of BM stimuli using an adapted visual search task. In Experiment 1, we examined whether listening to temporally congruent footstep sounds, as compared with listening to incongruent sounds, would promote the search for point-light walker (PLW) targets embedded in a crowd of PLW distractors. If audiovisual congruency could facilitate the attentional selection of BM signals, it might enhance search performance (e.g., result in shorter reaction time or higher search accuracy) in the audiovisual congruent condition than in the incongruent condition. Besides, by adopting a no-sound condition as the baseline, we examined whether the congruent sounds would confer benefits and the incongruent sounds would cause impairments to the search relative to the baseline. Results from Experiment 1 revealed a significant congruency effect mainly driven by the facilitation of congruent sounds.

To further examine the roles of the critical BM cues in the observed effect, we removed the local motion cue (Experiment 2) and the global configuration cue (Experiment 3) from the visual stimuli, respectively, while keeping the low-level temporal correspondence of the audiovisual signals unchanged. If the temporal congruence of sounds and local BM cues plays an essential role in the crossmodal effect, we should expect to observe this effect when the local BM cue is present (Experiment 3) but not when it is absent (Experiment 2). Otherwise, if the global BM configuration is crucial to the crossmodal effect, we might observe the effect only when the global configuration is intact (Experiment 2) but not when it is deprived (Experiment 3). Finally, we removed both global and local BM cues (Experiment 4) to assess whether crossmodal facilitation arose simply from low-level audiovisual correspondence that remained identical in all the experiments. If so, the crossmodal effect should occur in Experiment 4; otherwise, such an effect might disappear in Experiment 4.

## Methods

### Participants

A total of 80 participants (45 female, mean age+*SD* = 21.6+2.2 years) took part in the study, 20 (11–12 females) in each Experiment. A two-tailed power analysis using G*Power (Faul et al., 2007) indicated that a sample size of at least 15 participants would afford 80% power to detect an audiovisual integration effect with a high effect size (Cohen’s *d* ≥0.8), based on the results of previous studies (Chamberland et al., 2016; Saygin et al., 2008). We have further increased the sample size to 20 per experiment to adequately detect the potential interactions across experiments. All participants reported normal hearing and normal or corrected-to-normal vision. They were naïve to the purpose of the study and gave informed consent according to procedures and protocols approved by the institutional review board of the Institute of Psychology, Chinese Academy of Sciences.

### Stimuli

#### Visual Stimuli

**Fig. 1:**
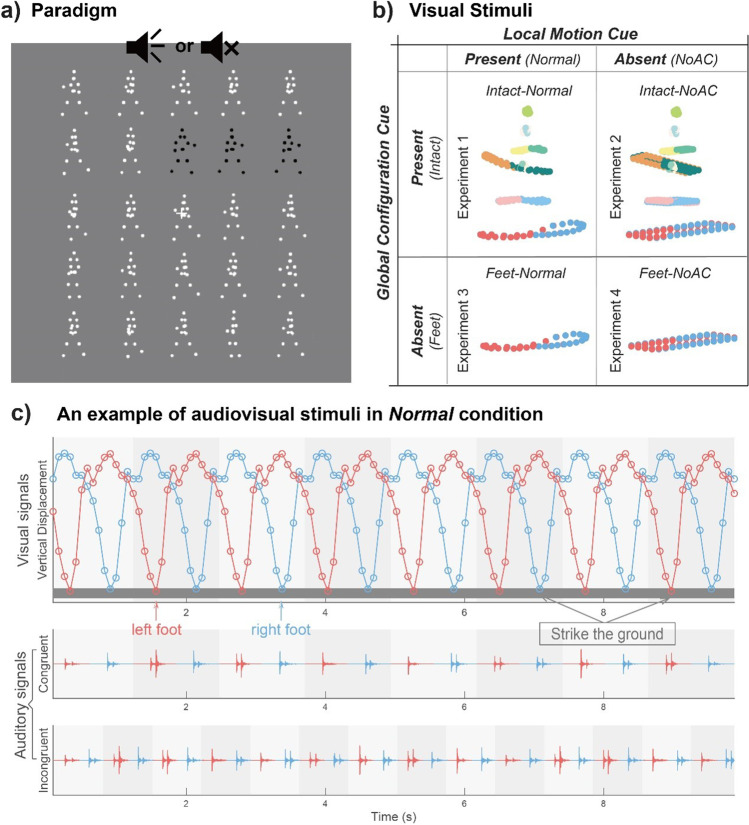
Illustrations of the experimental paradigm and stimuli. **a)** An example of the search display used in Experiment 1. The targets consisted of three collinear PLWs (shown in black for illustration only) whose walking direction differs from that of the distractors. Observers were required to judge whether the visual target appeared in a row or a column while listening to footstep sounds or no sound. The frequency of the footstep sounds could be congruent or incongruent with that of the gait cycle of the target walkers in different trails. **b)** Visual point-light stimuli used in Experiments 1–4. Individual joints of the stimuli were rendered in different colors to depict the motion trajectories of the joints within one gait cycle. All dots were white in the formal experiment. Different rows show BM stimuli with/without intact global configuration cues (*Intact/Feet*); different columns show BM stimuli with/without normal local kinematic cues (*Normal/NoAC*). **c)** An example of the visual and auditory signals in the *Normal* acceleration condition. Upper panel: The vertical displacement of the left foot (red line) and the right foot (blue line) changes nonlinearly over time within each gait cycle (highlighted by grey background colors), resulting in an acceleration pattern that occurs periodically at a constant frequency. The grey bar at the bottom of the figure (or the minimum y value of the dots) represents the position of the ground. Lower panel: waveforms of the temporally congruent and incongruent footstep sounds displayed on the same timeline. (Color figure online)

Figure 1b depicts the experimental design and the visual stimuli used in each experiment. In Experiment 1, the visual stimuli were normal PLWs (*Intact-Normal*) that consisted of 13 point-light dots attached to the head and the major joints of a human walker (Vanrie & Verfaillie, 2004). The temporal frequency of the walker is defined by the number of gait cycles (two steps) per second. In Experiment 2, we removed the vertical and horizontal acceleration cues from each limb-joint of the normal PLWs to disrupt the natural, gravity-compatible kinematic profile of BM (*Intact-NoAC*), generating point-light stimuli with intact global configuration but impaired local motion cues (Chang et al., 2018; Chang & Troje, 2009). In Experiment 3, we displayed feet-only sequences with normal acceleration profiles (*Feet-Normal*), given that such stimuli convey the most critical local motion information in BM while lacking intact global configuration (Bardi et al., 2014; Troje & Westhoff, 2006; Wang et al., 2014). In Experiment 4, we further removed acceleration cues from the feet-only sequences (*Feet-NoAC*) to disrupt both the local and global BM cues, creating nonbiological motion stimuli that were matched with BM only in low-level properties.

#### Auditory stimuli

Auditory stimuli were continuous footstep sounds (10 s) with a sampling rate of 44100 Hz. The sound sequences contain periodic impulses whose peak amplitudes occur around the points when the foot strikes the ground, with little variations in the waveforms of each step (Fig. 1c). We manipulated the duration interval between the time points when the two feet hit the ground to control the temporal frequency of the footstep sounds. Note that the auditory signals were always the same in all the experiments and were equally congruent with the visual stimuli regardless of the manipulation of accelerations, as we maintained the alignment between the sounds and the visual footstep events when removing the acceleration cues from the visual stimuli.

#### Stimuli presentation

All stimuli were displayed using MATLAB (MathWorks, Inc) together with PsychToolbox extensions (Brainard, 1997; Pelli, 1997). Participants sat 60 cm from the screen in a dim room, with their heads stabilized on a chinrest. The visual stimuli were displayed on a 37.5 cm × 30 cm CRT monitor with a resolution of 1,280 × 1024 pixels. The refresh rate was 85 Hz. The sounds were presented binaurally through headphones.

### Procedure and design

#### Experiment 1

Each trial began with a white fixation cross (0.6° × 0.6°) displayed at the center of the screen. Participants were required to maintain fixation on that cross throughout the trial. After 1,000~1,500 ms, a visual search display with 25 PLWs appeared (Fig. 1a). Participants were required to search for the target—three collinear PLWs with a predefined walking direction different from those of the distractors—among a 5 × 5 array and judge whether the target appeared in a row or a column by pressing different keys as quickly as possible while minimizing errors. Each PLW subtended approximately 0.84°×2.18°. The distance between the centers of every two PLWs was 2.71° in horizontal and 3.05° in vertical. The target-PLW walked at a higher or lower speed (i.e., at the temporal frequency of 1.42 Hz or 0.81 Hz) compared with the distractors. To ensure that any three aligned distractors would not walk at the same pace, we assigned two different frequencies to the distractors: 0.81 & 1.13 Hz when the target frequency was 1.42 Hz, and 1.42 & 1.13 Hz when the target was 0.81 Hz. The visually displayed PLWs were paired with footstep sounds, which had the congruent (1.42 Hz or 0.81 Hz) or incongruent (0.81 Hz or 1.42 Hz) frequency relative to the visual target. In addition to the audiovisually congruent and incongruent conditions, we adopted a no-sound condition as the baseline. There were 40 trials in each condition. We divided these trials into two baseline blocks and four sound blocks based on the frequency of the footstep sounds, with each baseline block present in between two sound blocks.

#### Experiments 2–4

Experiments 2–4 had the same procedures as Experiment 1, except for the visual stimuli (see the Visual Stimuli section for details), and we used a 4 × 4 stimulus array to control for task difficulty.

### Data Analyses

For each participant, correct trials with reaction time (RT) within 3 standard deviations from the mean were included in further analysis. The percentages of trials excluded from the analyses were 0.77% for Experiment 1, 0.31% for Experiment 2, 0.83% for Experiment 3, and 0.78% for Experiment 4. For each experiment, a one-way repeated-measures analysis of variance (ANOVA) was used to test the influence of three audiovisual conditions (congruent, baseline, incongruent) on reaction time and accuracy. In the cross-experiment analysis, to deal with the weakness of adopting a between-subject design in such analyses, we calculated a normalized audiovisual congruency effect to correct for individual variances in search performance across experiments. Meanwhile, considering the significant sound effect on both reaction time and accuracy in Experiment 1 and any potential speed–accuracy trade-offs in other experiments, we calculated the normalized audiovisual congruency effect based on the inverse efficiency (IE) scores that take into account both reaction time and accuracy (Ngo & Spence, 2012; Spence et al., 2001), as follows ($\frac{{\textrm{IE}}_{\textrm{Incongruent}}-{\textrm{IE}}_{\textrm{Congruent}}}{\left({\textrm{IE}}_{\textrm{Congruent}}+{\textrm{IE}}_{\textrm{Incongruent}}+{\textrm{IE}}_{\textrm{Baseline}}\right)/3}$). We also calculated the normalized benefit effect induced by congruent sounds ($\frac{{\textrm{IE}}_{\textrm{Baseline}}-{\textrm{IE}}_{\textrm{Congruent}}}{\left({\textrm{IE}}_{\textrm{Congruent}}+{\textrm{IE}}_{\textrm{Incongruent}}+{\textrm{IE}}_{\textrm{Baseline}}\right)/3}$) and the cost effect induced by incongruent sounds ($\frac{{\textrm{IE}}_{\textrm{Incongruent}}-{\textrm{IE}}_{\textrm{Baseline}}}{\left({\textrm{IE}}_{\textrm{Congruent}}+{\textrm{IE}}_{\textrm{Incongruent}}+{\textrm{IE}}_{\textrm{Baseline}}\right)/3}$) relative to the baseline in the similar way. Then, a two-way ANOVA, with global configuration (present vs. absent) and local motion (present vs. absent) cues as between-subjects factors, was conducted to compare each normalized sound effect (i.e., the congruency, benefit, or cost effect) across experiments.

## Results

### Experiment 1: Temporally congruent footstep sounds facilitate the visual search of BM signals

**Fig. 2:**
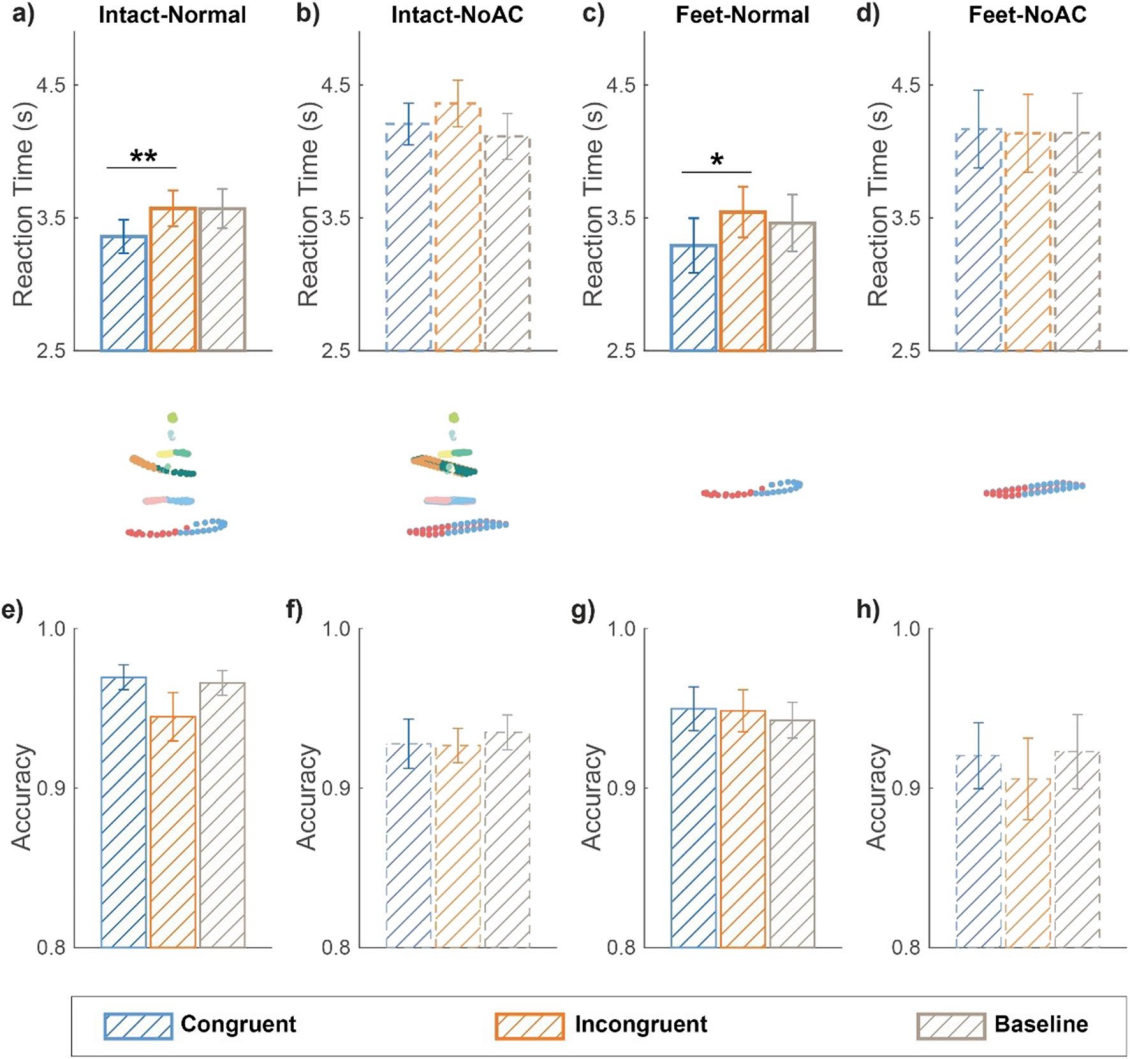
Mean reaction times **(a–d)** and accuracies **(e–h)** for different sound conditions in Experiments 1–4, where the BM stimuli have intact or not intact global configuration cues (Intact vs. Feet) and normal or disrupted local kinematic cues (Normal vs. NoAC). **p < .01, *p < .05, after Bonferroni correction. Error bars represent ±1 standard error of means. (Color figure online)

A repeated-measures ANOVA on RT revealed a significant main effect of sound conditions (congruent, incongruent, baseline; Fig. 2a), *F* (2, 38) = 4.864, *p* = .013, *η*_p_^2^ = 0.204. A post hoc test showed that RT for incongruent trials was significantly slower than that for congruent trials, *t*(19) = 3.621, *p*_uncorrected_ = .002, *p*_Bonferroni_ = .005, indicating the presence of a crossmodal facilitation effect. Moreover, compared with the baseline condition, congruent audiovisual signals marginally facilitated the visual search, *t*(19) = −2.358, *p*_uncorrected_ = .029, *p*_Bonferroni_ = .088, while incongruent audiovisual signals did not impair the performance, *t*(19) = 0.033, *p*_uncorrected_ = .974, *p*_Bonferroni_ = 1.000.

The same analysis was performed on accuracy. There was a significant main effect of sound conditions (Fig. 2e), *F*(2, 38) = 3.356, *p* = .045, *η*_p_^2^ = 0.150, and no significant difference was observed among the three conditions, except that the accuracy in the congruent condition was higher than that in the incongruent condition before Bonferroni correction, congruent vs. incongruent, *t*(19) = 2.266, *p*_uncorrected_ = .035, *p*_Bonferroni_ = .106; baseline vs. congruent, *t*(19) = −0.556, *p*_uncorrected_ = .585, *p*_Bonferroni_ = 1.000; baseline vs. incongruent, *t*(19) = 1.672, *p*_uncorrected_ = .111, *p*_Bonferroni_ = .333.

## Experiment 2: No crossmodal facilitation effect with disrupted local BM cues

To further determine the specific contributions of local and global BM cues to the observed audiovisual facilitation effect, we carried out Experiments 2–4. Research suggests that the specificity in visual BM perception is driven by gravity-compatible acceleration cues in the local BM signals (Chang & Troje, 2009; Troje & Chang, 2023; Troje & Westhoff, 2006; Vallortigara & Regolin, 2006). If these local kinematic cues are critical to triggering the audiovisual integration of BM information, we should expect that removing such cues would eliminate the crossmodal facilitation effect.

Results from Experiment 2 supported this hypothesis. When acceleration cues were removed, despite the significant main effect of sound condition (Fig. 2b), *F*(2, 38) = 3.763, *p* = .032, *η*_p_^2^ = 0.165, the difference between the incongruent and congruent conditions was no longer evident, *t*(19) = 1.656, *p*_uncorrected_ = .114, *p*_Bonferroni_ = .343. The RTs in the incongruent and congruent conditions were both greater than that in the baseline, yet only the incongruent condition significantly slowed the search performance relative to the baseline, *t*(19) = 3.085, *p*_uncorrected_ = .006, *p*_Bonferroni_ = .018. In addition, no significant effect of auditory conditions was observed on accuracy (Fig. 2f), *F*(2, 38) = 0.242, *p* = .786, *η*_p_^2^ = 0.013. These results suggest that local BM information is critical for the crossmodal search facilitation effect observed in Experiment 1.

## Experiment 3: Local motion alone could induce the audiovisual facilitation effect

In Experiment 3, we further explored whether local BM information alone was sufficient to explain the audiovisual facilitation effect by using the feet-only BM sequences, as the feet motions convey the most critical local BM cues but without intact global configuration (Chang & Troje, 2009; Troje & Westhoff, 2006). In line with the results from Experiment 1, the main effect of sound conditions was significant on RT (Fig. 2c), *F*(2, 38) = 4.257, *p* = .021, *η*_p_^2^ = 0.183. In particular, search performance was faster in the congruent condition relative to the incongruent condition, *t*(19) = −3.365, *p*_uncorrected_ = .003, *p*_Bonferroni_ = .010, suggesting that the crossmodal effect can occur even without intact global configuration information. Compared with the baseline condition, congruent sounds showed a tendency to speed up the search response, *t*(19) = −1.939, *p*_uncorrected_ = .067, *p*_Bonferroni_ = .202, and incongruent sounds did not yield an evident cost, *t*(19) = 0.824, *p*_uncorrected_ =.420, *p*_Bonferroni_ = 1.000. There was no significant main effect of sound conditions on accuracy (Fig. 2g), *F*(2, 38) = 0.283, *p* = .755, *η*_p_^2^ = 0.015.

## Experiment 4: The absence of facilitation effect without characteristic BM cues

If the BM-specific local motion cues (the characteristic acceleration patterns in the feet movements) account for the effect observed in Experiment 3, removing such acceleration cues from the feet motion sequences should eliminate the crossmodal facilitation effect despite that such manipulation did not alter the low-level temporal correspondence in the audiovisual stimuli. To test this assumption, we conducted Experiment 4. As expected, a repeated-measures ANOVA showed no significant main effect of the sound conditions on RT (Fig. 2d), *F*(2, 38) = 0.049, *p* = .953, *η*_p_^2^ = 0.003. In addition, no significant sound effect was observed for accuracy (Fig. 2h), *F*(2, 38) = 0.770, *p* = .470, *η*_p_^2^ = 0.039. These results show that temporally congruent sounds could not facilitate the search of non-BM signals, suggesting that the facilitation effects observed in Experiments 1 and 3 were specific to BM processing.

## Cross-experiment analysis: audiovisual facilitation hinges on local motion cues independent of global configuration

**Fig. 3:**
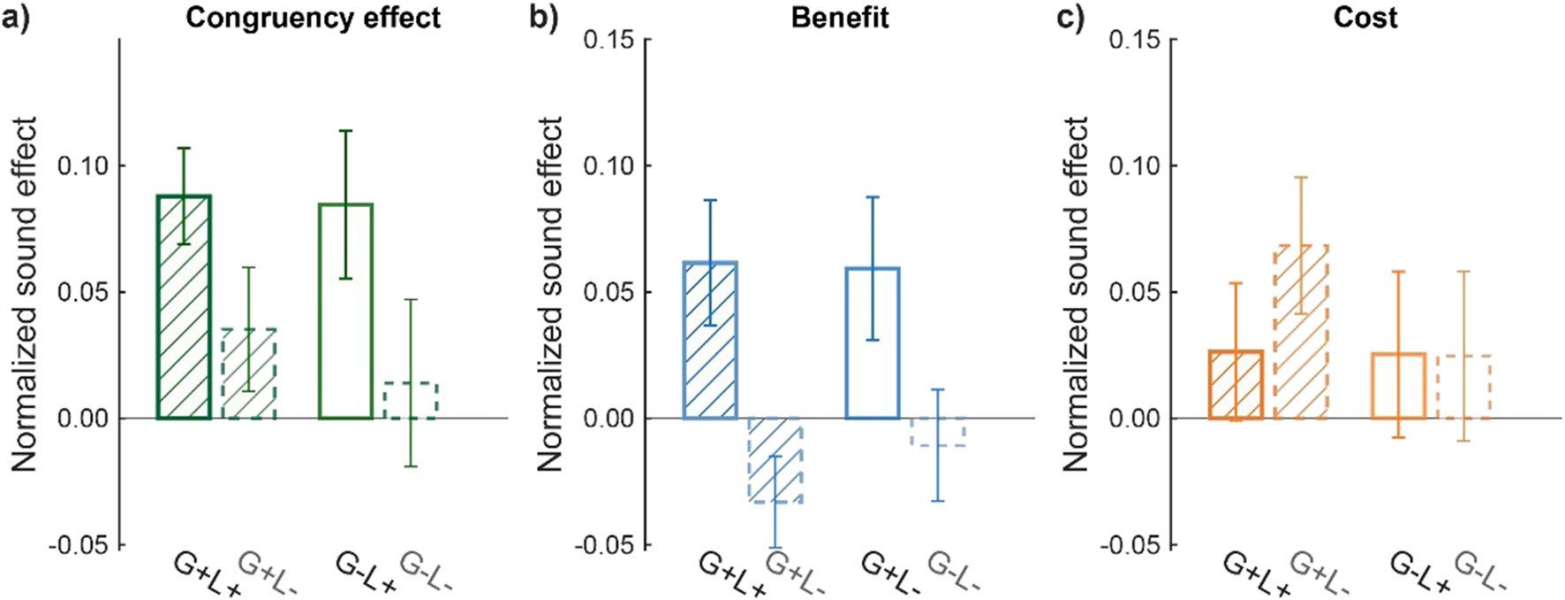
The normalized sound effects across experiments. **a)** The overall audiovisual congruency effect. **b)** The benefit effect of congruent sounds relative to the baseline. **c)** The cost effect of incongruent sounds relative to the baseline. G = Global configuration; L = Local motion; + : present; − : absent. Error bars represent ±1 standard error of means. (Color figure online)

To further compare the crossmodal effects across Experiments 1-4 and examine whether there is an interaction between local and global BM cues, we calculated a normalized audiovisual congruency effect (see Methods) to control for the influence of individual variances in search performance across experiments and performed a two-way ANOVA based on this normalized effect, with global configuration (present vs. absent) and local motion (present vs. absent) as between-subject variables. The analysis revealed a significant main effect of local motion (Fig. 3a), *F*(1, 76) = 5.223, *p* = .025, *η*_p_^2^ = 0. 064, but no significant main effect of global configuration cues, *F*(1, 76) = 0.207, *p* = .651, *η*_p_^2^ = 0. 003, or interaction between local and global cues, *F*(1, 76) = 0.112, *p* = .738, *η*_p_^2^ = 0. 001, suggesting that the audiovisual congruency effect mainly relies on the local motion cues independent of the global configuration.

We further performed ANOVA analyses on the normalized benefit effect of congruent sounds and the normalized cost effect of incongruent sounds relative to the baseline, in a way similar to that for the overall congruency effect (see Methods). The results for congruent sounds (Fig. 3b) were similar to that for the congruency effects, with a significant main effect of Local motion, *F*(1, 76) = 12.149, *p* < .001, *η*_p_^2^ = 0. 138, but no significant main effect of Global or Local × Global interaction (*p*s > .60). For the effects of the incongruent sound, neither the main effects of local and global BM cues nor their interaction was significant (Fig. 3c, *p*s > .46). Altogether, these results suggest that local motion rather than global configuration cues are crucial to the crossmodal search facilitation induced by congruent sounds.

## Discussion

Theories and knowledge about visual BM perception have developed for nearly half a century (Blake & Shiffrar, 2007; Hirai & Senju, 2020; Johansson, 1973). Whereas research on the audiovisual processing of BM information has emerged more recently, and the underlying mechanisms remain to be clarified (Arrighi et al., 2009; Brooks et al., 2007; Saygin et al., 2008; Schouten et al., 2011; Thomas & Shiffrar, 2010, 2013; van der Zwan et al., 2009). Here we found that the visual search for continuous dynamic BM signals in a crowded visual display was facilitated by temporally congruent yet spatially noninformative sounds.

Remarkably, these findings may reflect a domain-specific mechanism different from that underlying the crossmodal attentional facilitation of nonbiological artificial stimuli. The visual search facilitation driven by synchronized nonspatial auditory cues, known as the Pip and Pop effect, has been widely observed in studies using simple visual patterns (Chamberland et al., 2016; Gao et al., 2021; Staufenbiel et al., 2011; Van der Burg et al., 2008, 2010; Zou et al., 2012). Such crossmodal facilitation usually relies on the synchronization of auditory events with abrupt changes or onsets of the visual stimuli (e.g., Van der Burg et al., 2010). A plausible explanation is that integrating multisensory cues from meaningless information within a transient window may avoid spurious interactions of unrelated signals from a wider temporal range (Fujisaki et al., 2004; Van der Burg et al., 2008). These findings from nonbiological stimuli may partially account for the absence of crossmodal facilitation in the *NoAC* conditions, especially in Experiment 4, where the visual stimuli lacked biological features but still conveyed complex motion signals unfolding periodically without abrupt onsets or changes. However, critical biological features may modulate the time window of audiovisual integration and lead to crossmodal facilitation in normal BM conditions. Specifically, BM perception relies on the spatiotemporal summation and continuous tracking of rhythmic kinematic signals over time rather than at any single time point (Giese & Poggio, 2003; Neri et al., 1998; Shen et al., 2023). In addition, the multisensory processing of BM and non-BM has different properties in the temporal dimension (Arrighi et al., 2006; Saygin et al., 2008), and the audiovisual integration of BM depends not only upon timing but also on meaningful associations, like those between natural footstep sounds and motion (Arrighi et al., 2009; Thomas & Shiffrar, 2013). These findings, together with our observations, indicate that a mechanism based on both temporal correspondence and biological associations may underlie the audiovisual integration of BM information, thereby specifically facilitating the search for BM signals.

We further isolated the contributions of two fundamental BM cues (i.e., the global configuration and the local motion) to the crossmodal search facilitation effect in a series of experiments. We found that the facilitation effect was evident no matter whether the global configuration cue was present (Experiment 1) or absent (Experiment 3). By contrast, when we destroyed the local motion cue in limb movements, such effects disappeared regardless of whether the global configuration was intact (Experiment 2) or not (Experiment 4). These findings provide compelling evidence that local motion rather than global configuration is both necessary and sufficient for triggering the audiovisual binding of BM signals in a dynamic context and is responsible for the enhanced visual search performance. Moreover, they enrich our understanding of the dissociable roles of the global configuration and local motion cues in BM perception (Hirai & Senju, 2020). Previous research suggests that the dissociation may stem from the anatomically and functionally distinct neural responses to the form and motion cues in BM stimuli (Jastorff & Orban, 2009; Vaina et al., 2001; Vangeneugden et al., 2014), as well as the different genetic origins for local and global BM processing (Wang et al., 2018). In particular, researchers speculate that a phylogenetically evolved neural mechanism tuned to the local BM cue may act as a life motion detector to help direct attention to these signals in humans and animals (Lemaire & Vallortigara, 2022; Troje & Chang, 2023). The current findings suggest that the function of this life-detection system may extend to the multisensory perception of BM information. More specifically, the local BM cue may interact with the temporally congruent auditory cues to enhance the salience of life motion signals and facilitate attentional selection in a crossmodal situation.

Functional neuroimaging studies have demonstrated that both auditory and visual BM stimuli could selectively activate the posterior superior temporal sulcus (pSTS) (Bidet-Caulet et al., 2005; Grossman & Blake, 2001). The pSTS also plays a crucial role in combing auditory and visual BM signals (Meyer et al., 2011) and is sensitive to audiovisual temporal correspondence (Noesselt et al., 2007). Given its significance to unimodal and multimodal BM processing, the pSTS is a possible neural substrate for the BM-specific audiovisual facilitation effect observed in the current study. Moreover, researchers propose that the processing of local BM cues involves subcortical regions, such as superior colliculus, pulvinar, and ventral lateral nucleus (Chang et al., 2018; Troje & Westhoff, 2006). Whether subcortical neural substrates mediate the predominant role of local BM cues in the audiovisual integration of BM signals is a question that warrants further research. Besides, BM stimuli convey rhythmic structures (i.e., gait cycles) akin to that embedded in continuous human speech. Recent electroencephalogram (EEG) and magnetoencephalography (MEG) studies have shown cortical tracking of rhythmic linguistic structures associated with language perception and comprehension (Ding et al., 2016, 2017; Keitel et al., 2018). Future studies could verify whether similar neural mechanisms underlie the visual, auditory, and multisensory processing of BM information, as well as the crossmodal attentional facilitation of BM signals.

## Data Availability

All data generated during the current study are made available online (http://ir.psych.ac.cn/handle/311026/42953), and materials used during the study are available from the corresponding author upon request.
